# Updates on clinical trials evaluating the regenerative potential of allogenic mesenchymal stem cells in COVID-19

**DOI:** 10.1038/s41536-021-00147-x

**Published:** 2021-06-30

**Authors:** Dhavan Sharma, Feng Zhao

**Affiliations:** Department of Biomedical Engineering, Texas A&M University, College Station, TX United States

**Keywords:** Viral infection, Mesenchymal stem cells

## Abstract

Severe acute respiratory syndrome coronavirus 2 (SARS-CoV-2) has infected nearly 118 million people and caused ~2.6 million deaths worldwide by early 2021, during the coronavirus disease 2019 (COVID-19) pandemic. Although the majority of infected patients show mild-to-moderate symptoms, a small fraction of patients develops severe symptoms. Uncontrolled cytokine production and the lack of substantive adaptive immune response result in hypoxia, acute respiratory distress syndrome (ARDS), or multiple organ failure in severe COVID-19 patients. Since the current standard of care treatment is insufficient to alleviate severe COVID-19 symptoms, many clinics have been prompted to perform clinical trials involving the infusion of mesenchymal stem cells (MSCs) due to their immunomodulatory and therapeutic properties. Several phases I/II clinical trials involving the infusion of allogenic MSCs have been performed last year. The focus of this review is to critically evaluate the safety and efficacy outcomes of the most recent, placebo-controlled phase I/II clinical studies that enrolled a larger number of patients, in order to provide a statistically relevant and comprehensive understanding of MSC’s therapeutic potential in severe COVID-19 patients. Clinical outcomes obtained from these studies clearly indicate that: (i) allogenic MSC infusion in COVID-19 patients with ARDS is safe and effective enough to decreases a set of inflammatory cytokines that may drive COVID-19 associated cytokine storm, and (ii) MSC infusion efficiently improves COVID-19 patient survival and reduces recovery time. These findings strongly support further investigation into MSC-infusion in larger clinical trials for COVID-19 patients with ARDS, who currently have a nearly 50% of mortality rate.

## In the midst of the global pandemic

Defined as “the first pandemic of the 21st century” by World Health Organization (WHO)^1^, the coronavirus disease has spread worldwide and infected people of nearly every country through travel and community-based contacts within six months of its outbreak^2,3^. After its major outbreak in December 2019 in Wuhan (Hubei, China)^1^, WHO named the etiological agent of coronavirus disease-2019 (COVID-19) as severe acute respiratory syndrome coronavirus 2 (SARS-CoV-2) on February 11th, 2020^4,5^. SARS-CoV-2 belongs to the same virus family as severe acute respiratory syndrome coronavirus (SARS-CoV) and Middle East respiratory syndrome coronavirus (MERS-CoV), and is classified under the family *Coronaviridae*, order *Nidovirales*^4,6^. According to the phylogenetic clustering, coronaviruses are categorized in *alpha*, *beta*, *gamma,* and *delta* subgroups^7^. Among these, *alpha-* and *beta-*coronaviruses (including human coronaviruses HCoV-229E, HCoV-NL63, HCoV-OC43, and HCoV-HKU1) infect mammals, while *gamma* and *delta* coronaviruses primarily infect birds^6,8^. One of the major factors that make the COVID-19 outbreak uncontrollable is the *“varied severity” of* symptom manifestation among individuals with similar age and physiological conditions. This means a small population of infected patients (~2–5%) show incredibly severe symptoms, whereas others manifest moderate-to-mild symptoms or even seem asymptomatic but still are virus carriers^9^. Usually, COVID-19 patients are categorized under three major classifications indicating the extent and progression of the infection. These include (i) mild symptoms (~80% of the infected population): patients have a benign infection, minor or nonspecific symptoms, and will not progress to severe disease, (ii) moderate symptoms (~15% of the infected population): patients have overt pneumonia with/without hypoxia, localized inflammation, and will require hospitalization, and (iii) severe symptoms (~5% of the infected population): patients show systemic inflammatory response syndrome (SIRS), systemic hyper inflammation, ARDS, and require invasive/noninvasive mechanical ventilation and critical care management with 1–2% risk of fatal outcome (Fig. 2a)^10^. Although SARS-CoV-2 can infect any individual aged from a few weeks to over 90 years old, the highest population was averaged at 55.5 years of age, with higher mortality rates in elderly individuals than younger people^11,12^. The risk factors for symptoms that will require critical care include male gender, over 60 years of age, active/historical smoking, and presence of underlying conditions such as cardiovascular disease, chronic pulmonary disease, and diabetes^13^. By the end of February 2021, nearly 15 months after the COVID-19 outbreak, around 118 million individuals have been infected and more than 2.6 million have died worldwide (covid19.who.int/). In the United States alone, ~28 million individuals have been infected and more than half million have died (covid19.who.int/). Extensive research has been done in the past year with an unprecedented speed and accuracy to unveil its key structural and functional features at the molecular level, in order to meet the urgent needs of vaccine development, effective standard of care therapeutics, and novel regenerative medicine-based adjuvant therapies. Although 300 million vaccine doses have been administered worldwide with notable safety outcomes, MSC infusion is being tested in several clinical trials to determine its immunomodulatory and regenerative potential to alleviate severe COVID-19 symptoms especially in an elderly population with underlying conditions.

## Recent findings in the structural and functional features of SARS-CoV-2 and the mechanism of severe disease progression

### Molecular features of SARS-CoV-2: digging deeper

The SARS-CoV-2 virion is pleomorphic and enveloped with a diameter of approximately 80–120 nm. Its 26–32 kb sized genome shows a 79.0% and a 51.8% similarity with the SARS-CoV and the MERS-CoV virus, respectively. The nucleotide sequence of SARS-CoV-2 very closely resembles (87.6–89.0%) that of the bat-origin SARS-like coronavirus (bat-SL-CoVZC45), which makes the bat a likely host of SARS-CoV-2 virus^14^. It has been observed that multiple lineages of pangolin coronavirus have high similarities to SARS-CoV-2, suggesting pangolin as another possible host in the emergence of new coronaviruses and their zoonotic transmission^15^. Similar to SARS-CoV and MERS-CoV, SARS-CoV-2’s positive-sense RNA genome codes consist of four structural proteins: envelope protein (E), nucleocapsid protein (N), spike glycoprotein (S), and membrane matrix protein (M)^16^. Upon the virions’ entry inside the host cell, N proteins unwrap the viral genome to be translated by the host’s ribosomes, in order to form more viral proteins^16^. M proteins, with a triple-spanning transmembrane region, are most abundant at the virion membrane, which allows the binding and transfer of the viral genome and N proteins through the host’s phospholipid membrane^17^. S proteins are of key importance as they engage virus particles on a specific host cell receptor, named angiotensin-converting enzyme 2 (ACE-2)^18^. S proteins are trimeric and contain two domains. The upper globular domain has an ACE-2 receptor-binding site that engages and initiates virion entry into the cell. This receptor binding domain has the highest sequence variability among coronavirus genomes. The lower fusion domain is highly conserved among coronaviruses and contains hydrophobic fusion peptide that draws the host’s and the virion’s lipid bilayers close enough to initiate the fusion.

**Fig. 1:**
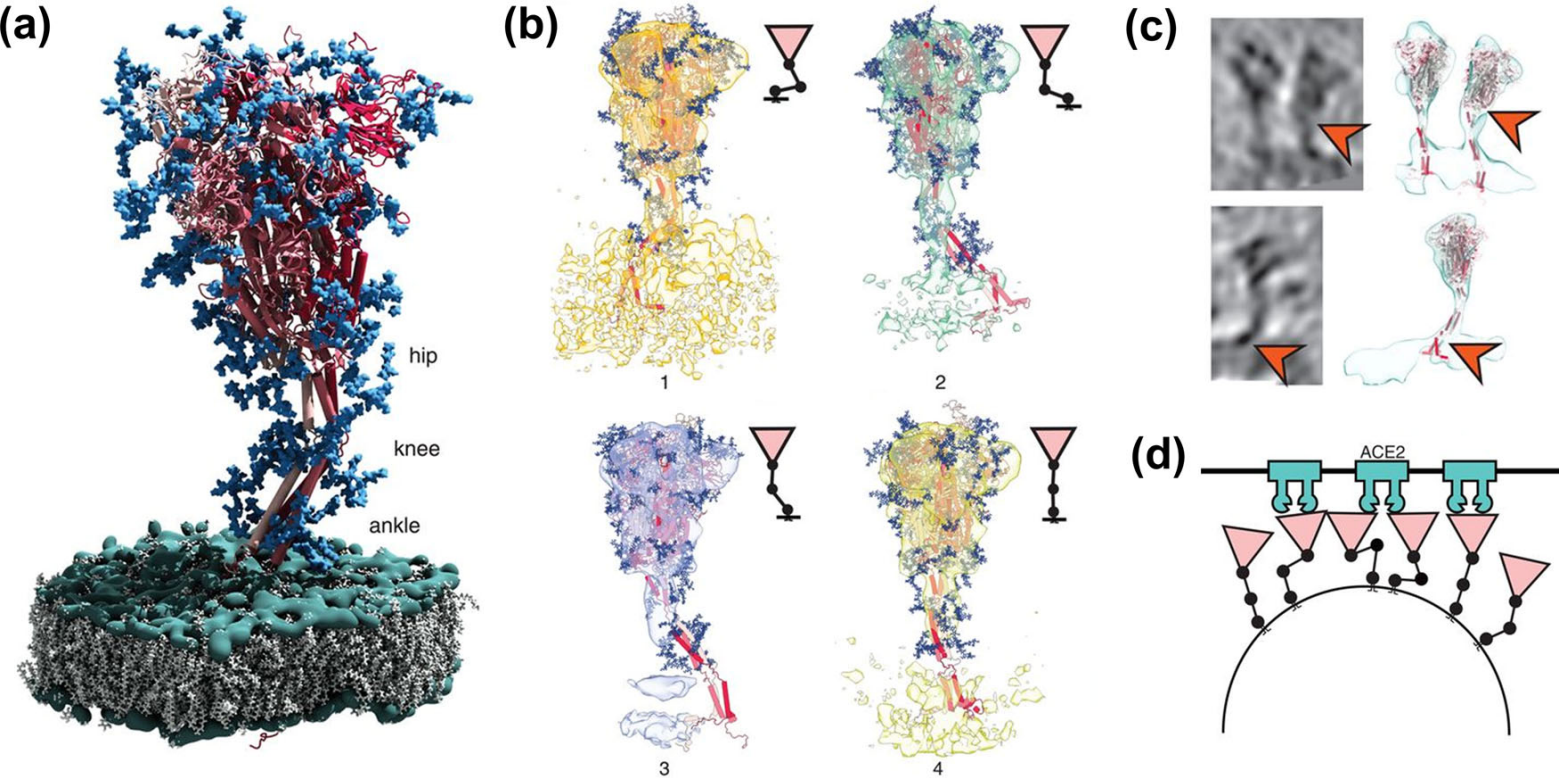
In situ structural analysis of SARS-CoV-2 spike protein. (from^19^. Reprinted with permission from AAAS). **a** Positions of the three flexible (hip, knee, and ankle) hinges of S protein. The model exhibits three individual chains of S protein (red), their N-glycosylation (blue), phosphates (green), and lipids of ER-like membrane (grey). **b** Molecular dynamics simulations show different distances of the head from the membrane indicating a shorter distance with a stronger bending and vice-versa. **c** Superimposed slices of actual tomograph and surface rendered snapshot of molecular dynamics simulations indicate consistency between structural modeling and tomographic data (orange arrowheads), (**d**) An illustration indicating hypothetical docking between multiple S heads and ACE-2 receptors facilitated by multiple hinges.

In a recent study, a combinatorial approach of cryo-electron tomography, subtomogram averaging, and molecular dynamics simulations have revealed structural features of the stalk part of S protein and how its conformational variability regulates viral attachment to the ACE-2 receptor^19^. According to the molecular dynamics simulation, the S head remains stable, while the stalk shows pronounce hinging motions at three distinct junctions: (i) between S head and the upper leg (hip joint), (ii) between the upper and lower legs (knee joint), and (iii) between the lower leg and the transmembrane domain (ankle joint) as shown in Fig. 1a. These simulations showed consistency with the leg segmentations observed in tomograms (Fig. 1b, c). The bending of the S stalk at different hinges provides the necessary flexibility to connect the heavily tilted S heads with the viral membrane. Importantly, it might also allow the S head to engage with increased avidity to the relatively flat host cell surface as illustrated in Fig. 1d^19^. The binding of S protein with ACE-2 receptor results in a proteolytic cleavage by a cellular transmembrane protein, known as transmembrane protease serine 2 (TMPRSS-2), that exposes the fusion peptide, a hairpin structure, which gets embedded in the membrane of the target cell and pulls the cellular and viral membranes closer for fusion. ACE-2 has been detected on the goblet and ciliated epithelial cells of the upper airway, Type II alveolar cells of the lower respiratory track, and pulmonary vasculature. In addition to the respiratory system, *ACE2* is widely expressed in the cells of vasculature, heart, gastrointestinal track, liver, kidney, central nervous system, and eyes^20^. The diverse ACE2 expression pattern in multiple tissues accounts for the secondary complications in COVID-19, including ARDS, cardiac injury, arrhythmia, acute kidney injury, and multiple organ dysfunction syndromes^21^. Individuals with diabetes, hypertension, or on ibuprofen medications are identified at higher risk for developing severe symptoms, as they have increased expression of ACE2 on their lung epithelial cells^22^. In addition to ACE2 and TMPRSS-2, the CD147 receptor has also be reported to be involved in mediating the cellular entry of SARS-CoV-2^23^. Recently, Wang et al. have reported a direct interaction between CD147 and S protein that mediates virus entry via endocytosis^24^. In their study, it was observed that the blocking of CD147 by Meplazumab successfully inhibited viral replication, while CD147 overexpression promoted viral infection, indicating the importance of CD147 in SARS-CoV-2 infection^24^. In a recent review, Machhi et al. have explained detailed mechanisms of the viral entry into a host cell, the molecular machinery that regulates genomic replication, transcription, and translational of viral components, and the mechanism that regulates the SARS-CoV-2 assembly inside the host cell and its exocytosis^25^.

### Mechanism of severe disease progression in COVID-19: influence of cytokines in shaping adaptive immune response

**Fig. 2:**
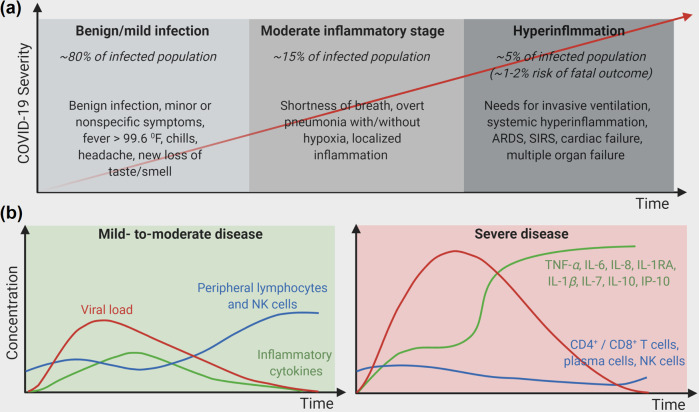
COVID-19, a disease with “varied severity” in symptoms. **a** Manifestation of symptoms with an increase in disease severity, (**b**) Comparison of viral titer, levels of peripheral lymphocytes, and key inflammatory cytokines over time in mild-to-moderate vs. severe COVID-19 patients.

During the acute infection phase, COVID-19 patients exhibit elevated levels of erythrocyte sedimentation rate (ESR), C-reactive protein (CRP), ferritin, serum amyloid A, as well as hypercytokinemia with elevated circulating cytokines including interleukin (IL)-1*β*, Interleukin-1 receptor antagonist (IL-1RA), tumor necrosis factor (TNF)-*α*, soluble IL-2 receptor alpha (sIL-2Rα), IL-6, IL-10, IL-17, IL-18, interferon (IFN)-*γ*, macrophage colony-stimulating factor (M-CSF), macrophage inflammatory protein (MIP)-1a, granulocyte colony-stimulating factor (G-CSF), interferon-gamma induced protein-10 (IP-10), monocyte chemoattractant protein (MCP)-1 and MCP-3. Among these, IL-1RA, IL-1*β*, TNF-*α*, IL-6, IL-7, IL-10, and IP-10 have been recognized as discriminative markers to identify mild-to-moderate or severe diseases (Fig. 2b)^26^. The molecular signature analysis of bronchoalveolar lavage fluid reveals that COVID-19 patients overexpress neutrophil recruiting mediators (CXCL1, CXCL2, CCL2, CCL7, and CXCL8) and the attractants of mainly innate immune cells (CCL2, CCL3, CCL4, CCL7, CCL8, CCL20, CXCL6, and CXCL11), resulting in COVID-19 a pulmonary centric disease^27^. Among these cytokines, type-I IFN, produced by plasmacytoid dendritic cells (pDCs), plays a pivotal role to regulate viral clearance, inhibits viral replication by RNA degradation, and triggers an adaptive response and tissue repair. It has been observed in multiple studies that mild-to-moderate patients had high type-I IFN response between 8–12 days of infection, while severe patients showed strikingly reduced regulation of IFN-stimulated genes^28,29^. During the first wave of hypercytokinemia, the increased expression of TNF-*α*, IL-6, and IL-10 prevents T lymphocytes’ recruitment and proliferation and even promotes their apoptosis, leading to T cell exhaustion. In a cohort involving 244 patients at Mount Sinai Health System, NY, USA, it was observed that severely diseased patients had significantly higher levels of TNF-*α*, IL-6, and IL-8, suggesting that the combination of these three cytokines can be used as a strong and independent predictor of patient survival^30,31^. In another study performed in Wuhan, China, the cytokine profile of critically ill patients showed higher levels of IL-2, IL-7, IL-10, TNFα, G-CSF, IP10, MCP-1, and MIP-1a, along with strikingly higher IL-6 levels in nonsurvival patient group^32^. Autopsy findings in COVID-19 patients have revealed that the secondary lymphoid tissues in these patients are destroyed with obvious spleen and lymph node atrophy, in addition to reduced numbers of CD4^+^ and CD8^+^ lymphocytes present in these tissues^33^. In a study involving 123 COVID-19 patients with lymphocytopenia, it was observed that the mild and the severe group showed a 28.43% and a 61.9% reduction in CD8^+^ T cell population, respectively; besides their corresponding 34.31% and 47.62% reduction in natural killer (NK) cell population (Fig. 2b)^34^.

In COVID-19, cytokine storms usually originate in a focal area and spread across the body via circulation^35^. A majority of the studies consistently proved that the primary cytokine storm induced after the viral infection is regulated mainly by resident alveolar macrophages, epithelial cells, and endothelial cells, while the secondary cytokine storm is regulated by infiltrated monocytes/macrophages and multinucleated giant cells but very few T lymphocytes. Since lymphocytes do not express ACE2 receptors, it has been speculated that they are destroyed by cytokine storms, precluding the possibility to generate a strong adaptive immune response^35^. Conclusively, severe COVID-19 patients have (1) abnormal T cell function, (2) an inefficient clearance of infected/activated macrophages, (3) escalated viral replication/dissemination, and (4) activation of more macrophages by IL-18 and IFN-*γ* feed-forward loop, which results in multiple cytokine release, hemophagocytosis, coagulopathy, and ARDS. In an excellent comprehensive review, Jamilloux et al., have explained the effect of this varied cytokine expression in shaping the innate and adaptive immune response among mild-to-moderate or severe COVID-19 patients^36^.

## Therapeutic strategies for severe COVID-19 and a need for MSC-based adjuvant therapy

The criteria that define severe or critically ill patients include but are not limited to are respiratory rate ≥30 times per minute, ≤93% of pulse oxygen saturation at rest, ≤300 mmHg of partial pressure of oxygen, and a fraction of inspired oxygen ratio (PaO_2_/FiO_2_), and a requirement for mechanical ventilation/shock^37^. Current non-vaccination treatment options for COVID-19 include antiviral drugs, anti-inflammatory drugs, monoclonal antibodies, and convalescent plasma therapy. The antiviral drugs are further categorized as: (i) RNA polymerase inhibitors (Remdesivir, Favipiravir), (ii) Protease inhibitors (Lopinavir-Ritonavir), (iii) virus entry-cell fusion inhibitors (Umifenovir, Camostat), and (iV) blockers for interleukin receptors and downstream signaling pathways (Anakinra and Tocilizumab, which blocks IL-1 and IL-6 receptor, respectively). Anti-inflammatory drugs such as Ruxolitinib and Baricitinib blocks JAK signaling and promotes immune suppression, while glucocorticoids suppress the inflammatory response. Among these drugs, Remdesivir has been approved by the FDA on October 22nd 2020^38^, while the rest of the drugs are under clinical trials. Currently, monoclonal antibodies that inhibit viral entry into the host cells are approved under Emergency Use Authorization (Bamlanivimab, Casirivimab, and imdevimab)^39–43^. Current vaccine platforms include (1) inactivated viruses, (2) live attenuated viruses, (3) genetically engineered nucleic acids (RNA and DNA) against S protein, (4) recombinant protein, and (5) viral vector-based vaccine. Formulations, immunological properties, and delivery of these vaccine platforms have been summarized in a recent review^39^. Increasing evidence from clinics have indicated that the critically ill and especially elderly patients require effective adjuvant therapies besides standard of care treatments to reduce mortality rate and improve recovery. The present regenerative medicine-based adjuvant therapies that have been accepted by clinics or clinical trials include infusion of convalescent plasma, and transplantation of MSCs and MSC-derived extracellular vesicles (EVs). The importance of convalescent plasma treatment has been previously established for effectively treating infectious diseases, including severe ARDS caused by SARS-CoV, MERS-CoV, Ebola, and Swine flu (A/H1N1)^44^. The convalescent plasma carries neutralizing antibodies, which efficiently and rapidly reduces viral load, eventually suppressing acute inflammation^44,45^. Although effective, the provision of convalescent plasma therapy drastically depends on the plasma collection program at the local demographic level from patients who have recovered from and are tested negative for COVID-19. Alternatively, leveraging regenerative paracrine secretion capability of allogenic MSCs as well as MSC-derived EVs via intravenous drip in COVID-19 can be proved as an effective “off-the-shelf” adjuvant therapy with rapid distribution capability^46,47^. A few reviews have been published in the past year summarizing the pre-clinical data and predicting the future of MSC therapy for critically ill COVID-19 patients^48–50^. However, in the current article, we have excluded single patient case reports and smaller pre-clinical studies and mainly focused on evaluating the safety and efficacy outcomes of the most recent and placebo-controlled phase I/II clinical studies that enrolled a large number of patients (between 10–100), in order to provide a statistically relevant and comprehensive understanding of MSC’s therapeutic potential to alleviate severe COVID-19 symptoms.

## Rational for selecting MSCs as an adjuvant therapy for COVID-19

**Fig. 3:**
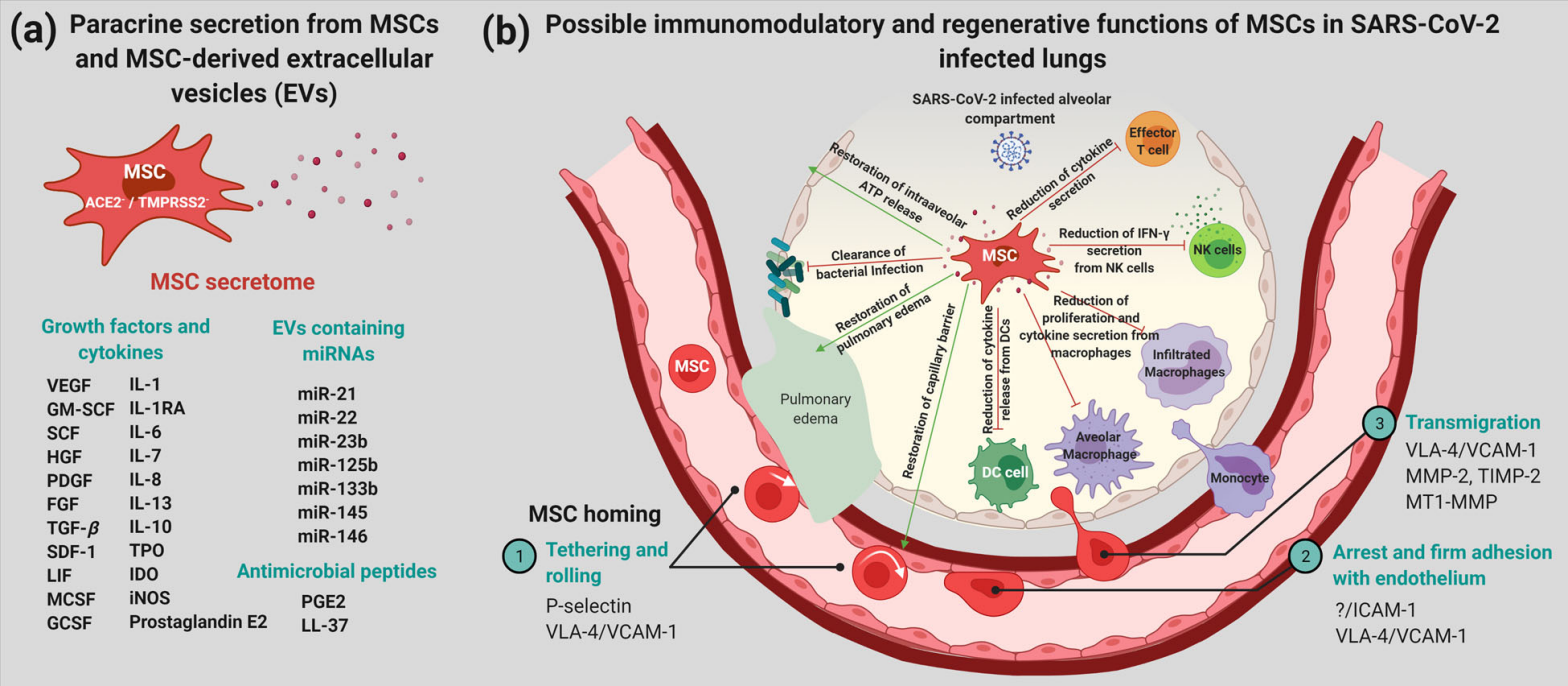
MSCs / MSC-derived EVs infusion in COVID-19 patients. **a** Paracrine factors secreted by MSCs and MSC-derived EVs, (**b**) Mechanism of MSC homing and possible immunomodulatory and regenerative functions of MSCs in the alveolar compartment of COVID-19 patients.

MSC is a preferred acronym that stands for a population of multipotent stem/progenitor cells, commonly known as mesenchymal stem cells, mesenchymal stromal cells, multipotent stromal cells, and mesenchymal progenitor cells^51,52^. MSCs can be isolated from various tissue sources, such as bone marrow, adipose tissue, peripheral blood, placenta, umbilical cord, amniotic fluid, and gingival tissues. They also have the excellent proliferative capability, and an intrinsic differentiation potential that has not been found in any other natural cell types^52^. MSC infusion into human patients begun since the year 1993 and has been reported as early as in 1995^53^. Since then, during the past 25 years, MSC infusion has exhibited an excellent safety profile in over 950 registered clinical trials and with over 10,000 patients, treated in a clinical setting^52^. MSC has powerful immunomodulatory and endogenous repair and regenerative properties. In the past, MSCs have been clinically tested for the treatment of graft versus host diseases, virus-associated immune abnormalities, and chronic injuries in human immunodeficiency virus, hepatitis B virus, and influenza virus^54^. MSC infusion has shown variable yet promising results in ARDS with viral^55^ or nonviral^56–58^ etiology through paracrine mechanisms including secretion of growth factors and cytokines as well as the release of EVs comprising exosomes and microvesicles^59^. The mass spectroscopy-based analysis has revealed that the EV cargo contains more than 850 unique gene products and more than 150 miRNAs that modulate immune responses as illustrated in Fig. 3a^60,61^.

It has been reported that MSCs are attracted to the site of inflammation following proinflammatory cytokine gradients and bind to the endothelium via a P-selectin-dependent manner. In a microvessel surrounding inflamed tissue, rolling MSCs interact with very late antigen-4 (VLA-4)/vascular cell adhesion protein-1 (VCAM-1) receptors that promote their firm adhesion on the endothelial cell surface^62^. Lastly, MSCs’ extravasation/trans-endothelial migration is regulated mainly by matrix metalloproteinase 2 (MMP2), membrane type I-matrix metalloproteinase (MT1-MMP), and tissue inhibitors of metalloproteinases-2 (TIMP-2)^63^ (Fig. 3b). MSC’s immune-regulation mechanism involves modulating the activation and effector function of innate and adaptive immune cells, suppressing lung-infiltrated immune cells, and enhancing the resolution of pulmonary edema^64^. Specifically, MSCs release GM-CSF, prostaglandin E2, keratinocyte growth factor, Interleukin (IL)-6, and IL-13 to facilitate the phagocytosis and alternative activation of alveolar macrophages. These factors also help to reduce the interferon IFN-*γ* secretion from NK cells and alter the cytokine secretion profile of dendritic cells (DCs)^37^. MSCs also release IL-10, transforming growth factor -*β* (TGF-*β*), and tryptophan catabolizing enzyme indoleamine 2,3-dioxygenase, which suppress the T cell proliferation and alter its cytokine secretion profile^65^. Besides immunomodulatory effects, MSCs can restore capillary barrier^66^, intra-alveolar ATP release^67^, and inhibit bacterial growth by secretion of the antimicrobial agent PGE2 and LL-37 peptide^68^ to reduce ARDS severity in lungs. The capability of MSCs to modulate immune responses becomes critical in situations where macrophages, DCs, NK cells, and T cells generate severe cytokine storms because of their faster proliferation. One of the earliest studies performed in January–February 2020 involving the infusion of clinical-grade hMSC in 7 severe COVID-19 patients indicated robust therapeutic and immunomodulatory effects of allogenic hMSCs. In this report, a 10× single-cell RNA-seq survey revealed that the MSCs were ACE2^−^/TMPRSS2^−^ and therefore were free from SARS-CoV-2 infection^69^. Besides, the Kyoto Encyclopedia of Genes and Genomes (KEGG) analysis indicated that MSCs were involved in antiviral pathways, which made them an ideal candidate for regenerative medicine-based therapeutic approach^69^. Several reviews have been published in the past few months, which have predicted how the MSC therapy benefited the COVID-19 patients and its possible mechanism of action^70–72^. Herein, we have reviewed the most recently published phase I/II clinical trials (Table 1) to further clarify (1) how safe it is to infuse allogenic MSCs in severe COVID-19 patients, and (2) how efficient MSCs are to alleviate severe COVID-19 symptoms including curtailing the cytokine storms. We believe that the critical evaluation of outcomes from these MSC based clinical trials as well as MSC infusion-associated challenges will be of particular importance for their application in COVID-19 treatment.

**Table 1:** Recently published phase I/II clinical trials testing safety and efficacy of MSCs or MSC-derived exosomes as an infusion product for the treatment of moderate to severe COVID-19 patients.

Clinical trial registration / Location	Infusion product	Study design	Number of patients enrolled	Key outcomes	Adverse event log	Ref.
NCT04269525 /China	Umbilical cord–derived MSCs	Control group not included	16	Improvement in oxygenation index and chest imaging; recovery of lymphocyte subset count and reduction in CRP and PCT post MSC infusion	None MSC infusion related adverse or allergic reactions; none delayed hypersensitivity or secondary infections	^73^
Approved by the Clinical Research Ethics Commissions of Taikangtongji Hospital, Wuhan, China	Umbilical cord–derived MSCs	Control group not included	31	Significant elevation in oxygenation index and lymphocyte counts; significant reduction in CRP, PCT, IL-6, and D-dimer levels	None MSC infusion related adverse or allergic reactions	^74^
ChiCTR2000031494/China	Umbilical cord–derived MSCs	Single-center, open-label, individually randomized trial	41	Faster recovery of oxygenation index and lymphocyte counts; significant reduction in CRP and IL-6 levels, significantly shorter lung inflammation absorption on CT imaging	None UC-MSC infusion related adverse or allergic reactions	^75^
NCT04252118/China	Umbilical cord–derived MSCs	Controlled and non-randomized	18	MSC infusion group showed improved oxygenation index, well controlled lung lesions and reduced trend of inflammatory cytokines (IL-6, IFN-γ, TNF-α, MCP-1, IP-10, IL-22, IL-1RA, IL-18, IL-8)	Two patients showed transient facial flushing with fever; one patient showed transient hypoxia; all of which were resolved within 24 h. Besides, no UC-MSC infusion-associated serious adverse events	^76^
NCT04288102	Umbilical cord–derived MSCs	Double-blind and placebo-controlled	100	UC-MSC group showed reduced total lung lesion proportion and solid component lesion, improved walking distance in a 6-minute walk test, but no significant difference in the peripheral lymphocyte subsets counts	One patient in MSC group showed grade 3 adverse event, which resolved spontaneously with conservative treatment. Besides, no MSC infusion related adverse or allergic reactions	^46^
NCT04355728	Umbilical cord–derived MSCs	Double-blind, placebo controlled, randomized trial	24	UC-MSC group showed reduced levels of pro-inflammatory cytokines within 6 days of treatment and resulted in a significantly improved	One patient with bradycardia required transient vasopressor treatment. Besides this, no serious adverse events were observed related to UC-MSC infusions	^77^
ChiCTR2000029990	Clinical grade MSCs	placebo-controlled	10	MSC infusion group showed significantly improved pulmonary function, increased peripheral lymphocytes count, dendritic cell population and reduced CRP and TNF-α levels.	No acute or delayed MSC infusion associated allergic reactions, hypersensitivity or secondary infections	^69^
Approved by The Spanish Medicines Agency (AEMPS) www.aemps.gob.es	Adipose tissue–derived MSCs	Control group not included	13	MSC infusion group showed reduction in CRP, lactate dehydrogenase (LDH), D-dimer and ferritin and improvement in B- and CD4 + /CD8 + T-lymphocyte counts.	Two patients died due to severe gastrointestinal bleeding unrelated to MSC therapy. Besides, no MSC infusion related adverse or allergic reactions	^78^
Approved by Christ Hospital’s (Jersey City, NJ) Institutional Review Board (IRB2020.01)	MSCs secreted exosomes (ExoFloTM)	non-randomized, open label cohort	24	ExoFlo^TM^ administration significantly improved oxygenation, absolute neutrophil, CD3 + , CD8 + and CD8 + lymphocyte counts and significantly reduced CRP, ferritin and D-dimer levels	4 patients deceased due to reasons unrelated to the ExoFloTM treatment. Besides, no ExoFloTM infusion related adverse or allergic reactions	^47^

## MSC infusion in COVID-19 patients: evaluating efficacies and therapeutic outcomes in the most recent clinical trials

### Umbilical cord derived MSCs (UC-MSCs)

MSCs can be isolated from several tissues including bone marrow, umbilical cord, placenta, adipose tissues, menstrual blood, etc. However, a majority of the clinical studies have selected allogenic umbilical cord-derived MSCs (UC-MSCs) as a readily available cell source that can be expanded to the clinical concentration very easily. In one of the earliest pilot study conducted by Feng et al., UC-MSCs were infused in a total of 16 (9 severe and 7 critically severe) COVID-19 patients between February 7th to April 1st, 2020^73^. In this study, clinical grade UC-MSCs suspended in normal saline were infused in 4 doses (at Day 1, 3, 5, and 7, with a concentration of 1 × 10^8^ cells/time point). Critically severe patients exhibited respiratory failure and required mechanical ventilation. Despite the small number of enrolled patients, improvement in oxygenation index, CRP, and procalcitonin (PCT) were observed after the UC-MSC infusion in both severe and critically severe patients. All of the patients showed improvements in CD4^+^ T cells, CD8^+^ T cells, and NK cell counts within 28 days of infusion, indicating the immunomodulatory effects of UC-MSCs. Although promising, this study lacks a control group, which makes it difficult to determine the therapeutic potential of UC-MSCs. But importantly, no acute infusion-associated allergic reactions were observed within two hours of the UC-MSC transplantation. Also, none of the patients displayed delayed hypersensitivity or secondary infections after the UC-MSC transplantation^73^. In another clinical study conducted between January to April 2020, a total of 31 patients (with a median age of 70 years) were given UC-MSCs infusion (1 × 10^6^ cells/kg of bodyweight) via intravenous drip^74^. Among these 31 patients, 19 patients required oxygen inhalation, 4 patients required noninvasive mechanical ventilation, 8 patients require invasive mechanical ventilation. Impressively, starting from the first infusion, 30 (96.8%) patients showed negative polymerase chain reaction (PCR) test results for SARS-CoV-2 within 10.7 ± 4.2 days. After the UC-MSC infusion, the PaO2/FiO2 level and lymphocyte counts were significantly elevated, while the CRP, PCT, IL-6, and D-dimer levels were significantly reduced in these patients compared to their levels before the UC-MSC infusion^74^. In accordance with the aforementioned study by Feng et al.,^73^ no UC-MSC-associated adverse allergic reaction was observed in this study^74^. Since the majority of the patients were given standard of care treatment besides the UC-MSCs infusion, and no control group was included in this study, it was difficult to statistically evaluate the therapeutic efficacy of UC-MSCs infusion^74^. To critically evaluate the effectiveness of UC-MSC infusion, a single-center open-label, individually randomized trial was performed (between February–March 2020) with 41 patients, which were divided into a standard treatment (provision of invasive or noninvasive oxygenation, antivirals, antibacterial drugs, and glucocorticoid therapy) group involving 29 patients and a standard treatment plus UC-MSCs infusion (2 × 10^6^ cells/kg) group, involving 12 patients^75^. In the UC-MSC group, all 12 patients were improved and discharged without the need for invasive ventilation. There was no incidence from severe to critically severe progression, and the 28-day mortality rate was zero. While in the control group, 4 patients deteriorated to a critical condition requiring invasive ventilation, of which, 3 died (28-day mortality rate 10.34%). Compared to the standard treatment control group, levels of C-reactive protein and IL-6 were significantly reduced from the third day of stem cell infusion in the UC-MSC group, which showed a faster recovery of lymphocyte count and oxygenation index to the normal range within 1 week of UC-MSC infusion. Computed tomography (CT) scan images indicated that the UC-MSC infusion improved CT scores, number of lobes involved, and consolidation that reflects reduced lung inflammation than the standard treatment control group. This study showed a clear improvement trend in patients who received UC-MSCs^75^. Most recently, Fu-Sheng Wang’s group has published a controlled, non-randomized phase I (enrolling 18 patients)^76^, and another randomized, double-blind, and placebo-controlled phase II clinical trial (enrolling 100 patients)^46^ to test the effectiveness of UC-MSC infusion in severe COVID-19. In their phase I trial, both control and treatment groups included 9 patients in each group (total n = 18)^76^. Among these patients, 1 patient in treatment group and 4 patients control groups required mechanical ventilation. The treatment group received 3 infusion of UC-MSCs (3 × 10^7^ cells/infusion) and exhibited declined IL-6 levels, improved PaO_2_/FiO_2_ ratio, and well-controlled lung lesions within 3 days of the cell infusion. Moreover, these patients showed a reduced trend of inflammatory cytokines including IFN-*γ*, TNF-*α*, IP-10, IL-22, IL-1RA, IL-18, IL-8, and MIP-1 within 14 days of UC-MSC infusion. Although two patients developed transient facial flushing with fever and one patient developed transient hypoxia at 12 h after the UC-MSCs transfusion, these events were resolved within 24 h, indicating the UC-MSC infusion was safe and well tolerated^76^. This phase 1 study was followed by a multi-center, randomized placebo-controlled phase 2 efficacy test with 100 severe COVID-19 patients, who received placebo (*n* = 35) or UC-MSC infusion (4 × 10^7^ cells/infusion, a total of 3 infusions) (*n* = 65) along with the standard of care treatments^46^. Among these patients, 44 (67.69%) patients from the treatment group and 23 (65.71%) patients from placebo group required supplemental oxygen. High-resolution chest CT images revealed that the UC-MSC group reduced the total lung lesion proportion and significantly lowered the solid component lesion compared to the placebo group. A 6 min walk test at 28th day posttreatment revealed that the UC-MSC group walked a longer distance (420 m) than the placebo group (403 m). No significant difference was observed in the peripheral lymphocyte subsets counts (CD4^+^ T cells, CD8^+^ T cells, B cells, and NK cells) and plasma markers between the two groups. Although one patient in the UC-MSC group showed a grade 3 adverse event, he/she recovered spontaneously under the conservative treatment, and no mortality was reported in this study^46^. Most recently Lanzoni et al., published a controlled, double-blind, randomized phase 1/2a clinical trial to determine the safety and efficacy of UC-MSC infusion in 24 patients with COVID-19 ARDS^77^. In this study, treatment (*n* = 12) and control (*n* = 12) group received two infusions (at day 0, day 3) of 100 ± 20 × 10^6^ UC-MSCs or vehicle solution (human serum albumin and heparin), respectively. Among these patients, 11 (46%) were receiving invasive mechanical ventilation, and 13 (54%) were on high flow oxygen therapy via noninvasive ventilation at the time of enrollment. One patient in UC-MSC treatment group died as a result of a failed endotracheal intubation, unrelated to the patient’s COVID-19 disease. At day 6 after UC-MSC infusion, a significant decline in the concentration of GM-CSF, IFNg, IL-5, IL-6, IL-7, TNFa, TNFb, PDGF-BB, and RANTES were observed in the UC-MSC group compared to control. Only adverse event occurred in UC-MSC treatment group in a subject with bradycardia, who required transient vasopressor treatment. Besides this, no serious adverse events were observed related to UC-MSC infusions indicating treatment safety for COVID-19 patients with ARDS. Overall, UC-MSC infusion resulted in significantly improved patient survival and recovery time^77^. Findings from these clinical trials indicated that UC-MSCs are safe to be used as adjunctive therapy along with the standard of care treatment for patients with moderate to severe COVID-19. Additionally, the United States Food and Drug Administration (US-FDA) has conditionally approved MSCs under ‘*expanded access compassionate use*’ for COVID-19. As a next step, a phase III trial was required to further evaluate the underlying mechanisms of UC-MSC treatment and its long-term effect on mortality and pulmonary disability in COVID-19.

### MSC derived from tissue sources other than umbilical cord

In one of the earliest pilot studies, Leng et al., tested early efficacy of clinical-grade MSC infusion (1 × 10^6^ cells/kg) in 7 COVID-19 patients along with the placebo treatment in 3 patients between January 23rd–February 16th, 2020^69^. The gene expression profile of MSCs indicated that they were ACE2^−^ and TMPRSS2^−^, thereby were safe from COVID-19 infection. All of the patients showed significant improvement in pulmonary function within 2 days of the MSC infusion. No acute or delayed MSC infusion-associated allergic reactions, hypersensitivity, or secondary infections were observed. Peripheral lymphocytes and CD14^+^ CD11c^+^ CD11b *mid* regulatory DC cell population was increased in these patients, whereas the CRP and TNF-*α* levels, overactivated cytokine secreting immune cell (CXCR3^+^ CD4^+^ T cells, CXCR3^+^ CD8^+^ T cells, and CXCR3^+^ NK cells) levels were dropped within 3–6 days of the MSC infusion compared to placebo group^69^. Although this study showed significant improvement with the infusion of clinical-grade MSCs, the tissue source of MSCs, from where they were isolated, was unclear^69^. In a recent proof of concept study, 13 severe COVID-19 patients requiring mechanical ventilation were infused with adipose tissue-derived mesenchymal stromal cells (AT-MSCs) besides the standard of care treatment, between April 3rd–22nd, 2020^78^. Among these, 10 patients received two infusions, 2 patients received a single infusion and 1 patient received 3 infusions with a median of 0.98 × 10^6^ cells/kg/infusion. Unfortunately, 2 patients died due to severe gastrointestinal bleeding unrelated to the MSC therapy. Besides this, no adverse events were observed related with AT-MSC infusion such as fever or worsening of respiratory or hemodynamic parameters. All 9 of the remaining patients (70%) showed clinical improvements, with a reduction in CRP, lactate dehydrogenase (LDH), D-dimer, and ferritin within 5 days of AT-MSC infusion. Moreover, 5 patients showed improvement in B- lymphocyte as well as CD4^+^ and CD8^+^ T-lymphocyte counts, and 7 (53%) were extubated within a median time of 7 days after the first AT-MSC infusion^78^. Besides UC-MSCs and AT-MSCs, several other MSC types are under investigation to test their therapeutic effects in COVID-19 and COVID-19 induced ARDS. The currently active clinical trials at various locations in the United States that involve different types of MSCs as an intervention product for COVID-19 are listed in Table 2. Interested readers are suggested to refer to a recently published review article for a worldwide list of stem cell-based active clinical trials that are “recruiting” or “not yet recruiting” COVID-19 patients^49^.

**Table 2:** Currently active phase II/III clinical studies involving MSCs isolated from different sources as an infusion product for the treatment of COVID-19 or COVID-19 induced acute respiratory distress syndrome (ARDS) at various locations in the United States.

Identifier	Status	Intervention product	Disease condition	Phase	Estimated number of enrollments	Locations	Estimated completion date
NCT04565665	Recruiting	Cord blood–derived MSCs	COVID-19, ARDS	I	70	M D Anderson Cancer Center, Houston, TX	April 30, 2021
NCT04399889	Recruiting	Cord tissue–derived MSCs	COVID-19	I/II	30	Duke Hospital, Durham, NC	July 31, 2021
NCT04466098	Recruiting	Mesenchymal Stromal Cells	COVID-19, ARDS	II	30	University of Minnesota, Minneapolis, MN; University of Pittsburgh, Pittsburgh, PA	December 1, 2021
NCT04629105	Recruiting	Longeveron MSCs (LMSCs)	COVID-19, ARDS	I	70	Miami VA Healthcare System, Miami, FL; University of Maryland Medical Center, Baltimore, MD; Wake Forest Baptist Medical Center, Winston-Salem, NC	July 31, 2025
NCT04397796	Recruiting	Bone marrow–Allo. MSCs	COVID-19	I	45	St. Francis Medical Center, Lynwood, CA	June 31, 2021
NCT04494386	Recruiting	Umbilical Cord Lining Stem Cells (ULSC)	COVID-19, ARDS, SARS-CoV-2	I/II	60	Sanford Research, Sioux Falls, SD	November 30, 2021
NCT04371393	Recruiting	Bone marrow–derived MSCs (remestemcel-L plus)	COVID-19, ARDS	III	300	Dignity Health, Gilbert, AZ; University of Southern California, Los Angeles, CA; Stanford University, Stanford, CA; and 18 more	April 30, 2022
NCT04524962	Recruiting	MSCs or MSC RNA– engineered to secrete a combination of DNases (Descartes 30)	COVID-19, ARDS	I/II	30	Brigham and Women’s Hospital, Boston, MA; University of Oklahoma Health Sciences Center, Oklahoma City, OK	September 25, 2022
NCT04389450	Recruiting	Allogeneic ex vivo expanded placental mesenchymal–like adherent stromal cells (PLX – PAD)	COVID-19, ARDS	II	140	University of California - Irvine, Irvine, CA; University of Southern California - Keck School of Medicine, Los Angeles, CA; and 11 more	March 31, 2022
NCT04348435	Enrolling by invitation	Hope Biosciences allogeneic adipose–derived MSCs	COVID-19	II	100	Hope Biosciences Stem Cell Research Foundation, Sugar Land, TX	April 30, 2021
NCT04362189	Active, not yet recruiting	Hope Biosciences allogeneic adipose–derived MSCs	COVID-19	II	100	River Oaks Hospital and Clinics, Houston, TX; United Memorial Medical Center, Houston, TX	October 31, 2020
NCT04349631	Active, not yet recruiting	Allogeneic adipose–derived MSCs	COVID-19	II	56	Hope Biosciences Stem Cell Research Foundation, Sugar Land, TX	December 31, 2020
NCT04573270	Completed	Intravenous MSC Injection (PrimePro)	COVID-19, Prophylaxis	I	40	Southern California Hospital at Culver City, Culver City, CA	September 1, 2020

### Preliminary safety and efficacy outcomes of MSC – derived extracellular vesicle (EV) infusion in COVID-19 patients

It has been believed that many, if not all, therapeutic benefits of MSCs can be attributed to their paracrine effects via release of EVs, phospholipid membrane–bound particles, rather than the actual cellular engraftment at the injury site^79,80^. EVs expressing common surface markers (CD9, CD63, and CD81) are generally classified as exosomes (40–150 nm in diameter), microparticles (50–1000 nm in diameter), or apoptotic bodies (500–2000 nm in diameter). EVs derived from a wide spectrum of MSC origins (including bone marrow, adipose tissue, peripheral blood, placenta, umbilical cord, amniotic fluid, and gingival tissues) are being investigated for regenerative medicine development, targeting several diseases^81^. There are several potential advantages of using EVs instead of MSC infusion^82^: (1) EVs can be administered directly to the lungs via intranasally or by inhalation instead of systemic delivery, (2) EVs eliminate the risk of uncontrolled cell proliferation, cellular senescence, and apoptosis, immune compatibility and the potential risk of transmission of infections, (3) Easier scale-up using a stir-tank or hollow fiber bioreactors to generate large quantities of EVs, which can be stored as an off-the-shelf product until required, (4) EVs can be engineered as a vehicle to payload therapeutic molecules into lungs, such as antiviral drugs or inhibitors that can temporarily block cellular endosomal pathway to prevent viral replication, (5) EVs can be decorated with viral spike proteins to block the cellular receptors to compete with cellular uptake of viruses.

In a recent non-randomized open-label cohort, 24 patients with severe COVID-19 were infused with ExoFlo^TM^, an allogenic bone merrow MSCs -secreted exosome product to test its safety and therapeutic efficacy^47^. After intravenous ExoFlo^TM^ administration, the patients’ clinical status improved with (1) a significant increase (192%) in oxygenation (PaO_2_/FiO_2_ ratio, *p* > 0.001) within 3 days of the treatment, (2) significant enhancement in absolute neutrophil as well as CD3^+^, CD4^+^ and CD8^+^ lymphocyte counts within 5 days of the treatment, and (3) significant decrease in CRP, ferritin and D-dimer levels post 5 days’ treatment. No adverse event was observed within 72 h of the ExoFlo^TM^ administration, although 4 patients deceased due to reasons unrelated to the ExoFlo^TM^ treatment^47^. Currently, Sengupta et al., are conducting a multi-center, placebo-controlled, randomized phase II trial to determine the efficacy of exosome delivery in 60 patients (ClinicalTrials.gov, Identifier: NCT04493242). Although ExoFlo^TM^ shows therapeutic benefits, Lim et al., has raised several questions regarding its production and application, including their derivation from BM-MSCs, biological activity, their actual concentration in the 15 mL of infusion dose, and the long term (>72 h) effects after the ExoFlo^TM^ administration^83^. These queries are still unanswered to date. More EV infusion-based clinical trials are required for a precise diagnostic evaluation of their therapeutic potential in curtailing severe symptoms of COVID-19.

## Concluding remarks and the next steps

The COVID-19 pandemic has spread worldwide very rapidly since its first outbreak in December 2019 and has resulted in ~2.6 million deaths worldwide. Since SARS-CoV-2 is a newly emerged virus, it is extremely challenging to develop effective drugs and vaccines as well as their delivery system. Several existing therapies are currently in practice or being tested under clinical trials to assess their efficacy for mild, moderate, or severe COVID-19 symptoms. Although, patients with mild-to-moderate symptoms can recover via conventional standard of care treatment, no robust treatment strategies are available for severely critical COVID-19 patients. This has prompted several clinical centers/hospitals to perform clinical trials involving the infusion of MSCs or MSC secreted EVs, due to their previously proven immunomodulatory and therapeutic properties. As summarized in this review, clinical outcomes obtained from these phase I/II trials have generated a very valuable record suggesting a strong therapeutic efficacy and safety of MSC-based adjuvant therapy alleviate severe COVID-19 symptoms. The next logical step is to refine and optimize the MSC products. It has been reported that MSCs isolated from heterogeneous tissue sources differ in their biological activity including proliferation rate, paracrine secretion profile, immunomodulatory activity, and anti-tumor activity. For example, AT-MSCs secrete higher levels of pro-angiogenic molecules including VEGF and MMPs compared to BM-MSCs. While UC-MSCs secrete higher levels of immunomodulatory factors including IL-6, IL-7, IL-10, TNF-*α,* and PDGF than BM-MSCs and AT-MSCs^84,85^. Verifying these results in human clinical trials requires an enormous amount of time and monetary investment. However, Emergency Use Authorization for MSC treatment by FDA during the COVID-19 pandemic has provided a remarkable opportunity to test and compare the various parameters of the MSCs infusion therapy. This includes comparing the efficacy of MSCs isolated from different tissue sources, their route of injection, optimum infusion concentration, needs for single or multiple doses, and time intervals between multiple MSC doses. Importantly, outcomes of the currently undergoing phase II/III clinical trials will generate an especially valuable comparison to test safety aspect and side effects among MSCs types isolated from different tissues. Another major translational hurdles to be overcome for MSC therapy is its high cost and current insurance policies. Usually, the cost of MSC therapy ranges between $5000–$50,000 depending on the location of the laboratory, location of patients, stem cell types, and their proliferative characteristics. In addition, Medicare does not cover the cost of MSC therapy in the United States currently, which significantly affects its successful clinical translation^86^.

With an increasing number of active clinical trials involving the infusion of MSC secreted EVs (which includes exosomes and microvesicles) in severe COVID-19 patients, it is important to investigate how EVs’ composition and production are influenced by different factors, such as stochastic process, clonal expansion, culture complexity and the state of MSC differentiation^52^. One of the known analogies in the EV field is: why bother buying the *cow* when you can directly buy the *milk*? - showcasing the difference between transplantation of MSCs *vs*. EVs. Although this analogy assumes EVs can potentially perform all the therapeutic and regenerative functions as efficiently as MSCs, it is mostly theoretical and yet to be proven in clinical settings. One promising possibility is, MSCs can be conditioned in vitro with particular cytokines to help the secretion of exosomes that have immunomodulatory property. In one such study, MSCs preconditioned with IFN-*γ* secreted exosomes that can block differentiation of T-helper 17 (Th17) cells, a subset of CD4^+^ T lymphocytes^87^. If EV delivery proves similar or better therapeutic/regenerative effectiveness and safety outcomes than MSC therapy, it will greatly alleviate the technological difficulties including large-scale production, quality control, storage, and delivery, to make it economical for entering clinics. Several active phase II/III clinical studies in the United States (Table 2) and all over the world will further generate robust efficacy and safety data, which will assist MSC-based therapies to enter clinics, not only to treat severe COVID-19 but also ARDS associated with SARS-CoV, MERS-CoV, Ebola, and Swine flu infection. Since intravenous MSC infusion can preferentially target lung tissues, this therapy has potential to treat ARDS-associated secondary trauma, microbial infections as well as pulmonary graft versus host diseases.
